# Pyrethroid Resistance in *Anopheles gambiae s.l.:* A Focus on Permethrin and Deltamethrin for Malaria Vector Control

**DOI:** 10.1590/0037-8682-0233-2025

**Published:** 2025-12-08

**Authors:** Oluwaseun Adegbola Adesoye, Adedayo Michael Awoniyi, Adedapo Oluwafemi Adeogun

**Affiliations:** 1 University of Abuja, Department of Biological Sciences, FCT, Nigeria.; 2 Universidade Federal da Bahia, Instituto de Saúde Coletiva, Salvador, BA, Brasil.; 3 Nigeria Institute of Medical Research, Molecular Entomology and Vector Control Research Laboratory, Yaba, Lagos, Nigeria.

**Keywords:** Anopheles gambiae s.l., Pyrethroid resistance, Permethrin, Deltamethrin, Vector control.

## Abstract

**Background:**

Malaria remains a major public health concern, and sub-Saharan Africa accounts for a significant proportion of global cases. A key challenge in malaria control is the increasing resistance of malaria vectors to commonly used insecticides, particularly pyrethroids. This study assessed the susceptibility of *Anopheles gambiae s.l.* to permethrin and deltamethrin in Zuba, located in the Gwagwalada Area Council of Abuja, Nigeria.

**Methods:**

Mosquito larvae were collected in June 2024 and reared under controlled insectary conditions until adulthood. Adult mosquitoes were then tested using the Centers for Disease Control and Prevention bottle bioassays, and exposed to both standard World Health Organization recommended doses (21.5 µg/bottle for permethrin and 12.5 µg/bottle for deltamethrin) and lower, sub-lethal concentrations. Mortality rates were monitored up to 24 h post-exposure, and the results were analyzed using one-way analysis of variance at a significance level of *P* < 0.05.

**Results:**

At the standard dose, permethrin induced a mortality rate of 84.4% (21.10 ± 0.50) after 30 min, significantly higher (*P* = 0.031) than that observed at 15.0 µg/bottle (18.00 ± 0.00, 72%). Complete mortality was achieved only after 35 min. Deltamethrin caused a mortality rate of 76% at 30 min (19.00 ± 0.30), with 100% mortality observed after 40 min (*P* = 0.04). No mortality was recorded for sub-lethal doses (≤0.8 µg/bottle) after 24 h (*P* = 0.061).

**Conclusions:**

These findings highlight the resistance levels of permethrin and deltamethrin in *An. gambiae s.l.* population in Zuba. Routine resistance surveillance and tailored mosquito control strategies are essential for sustaining mosquito control efforts in this region.

## INTRODUCTION

Malaria remains a major global public health concern, with an estimated 263 million cases and 597,000 deaths reported worldwide in 2023^1^. The disease is primarily transmitted by *Anopheles gambiae s.l.* ^2^ ^,^ ^3^, and children under the age of five remain the most vulnerable population, accounting for approximately 80% of all malaria-related deaths worldwide^4^. Sub-Saharan Africa bears the heaviest burden, contributing approximately 92% of global malaria cases and over 93% of malaria-related deaths^2^. Within the region, Nigeria stands out, accounting for nearly one-quarter of all malaria cases on the continent^5^ ^,^ ^6^, underscoring its critical role in both regional and global efforts against malaria.

Vector control remains a fundamental component of malaria prevention strategies in endemic regions^7^ ^,^ ^8^. The World Health Organization (WHO) recommends the use of chemical insecticides, particularly pyrethroids, such as deltamethrin and permethrin, for effective mosquito control^7^ ^,^ ^9^. However, the widespread emergence of insecticide resistance, defined by the WHO as physiological or behavioral changes that allow mosquitoes to survive exposure to standard insecticide doses lethal to susceptible individuals of the same species, following a specified diagnostic exposure time and post-exposure observation period^10^, is increasingly compromising the effectiveness of malaria control interventions. Resistance is typically confirmed when mosquito mortality falls below 90% at 24 h following a one-hour exposure to the discriminating concentration under standardized test conditions^10^. Historical entomological surveillance in Nigeria first identified the emergence of resistance to dicholorodiphenyl trichloroethane (DDT) in *An. gambiae s.l.* populations as early as the 1960s^11^. Despite this, DDT has been widely used in indoor residual spraying (IRS) campaigns for several decades, but it has now become increasingly ineffective due to the widespread development of resistance among key vector species, including *An. gambiae*, *An. coluzzii, Culex quinquefasciatus*, and *Aedes Aegypti* across various Nigerian states, including Oyo, Jigawa, and Lagos^12^ ^-^ ^14^. Resistance is predominantly driven by metabolic mechanisms, posing a significant challenge to existing vector control strategies^15^ ^-^ ^19^ and necessitating a strategic transition toward pyrethroid-based insecticides.

In response to the growing challenge of insecticide resistance, both the Centers for Disease Control and Prevention (CDC) and WHO recommend routine insecticide resistance surveillance as an essential component of malaria vector control programs in endemic regions^6^ ^,^ ^20^. Such surveillance enables early detection of resistance trends and facilitates timely adjustments to control strategies, thereby helping to preserve their effectiveness^21^ ^,^ ^22^. Similarly, consistent resistance monitoring generates valuable data to inform the development of innovative and sustainable vector management approaches^22^.

Despite these global efforts, there is, to the best of our knowledge, a lack of documented data on the insecticide resistance status of malaria vectors in Zuba, a semi-urban community in the Gwagwalada Area Council of Nigeria’s Federal Capital Territory (FCT), Abuja. Recent WHO reports emphasize the importance of localized insecticide resistance monitoring, considering that pyrethroid resistance is now widespread across sub-Saharan Africa, thereby threatening the continued effectiveness of key vector control tools such as insecticide-treated nets (ITNs) and IRS^1^ ^,^ ^2^. These reports advocate for district- and country-level entomological surveillance to generate context-specific evidence that can guide adaptive vector control interventions. In line with this guidance, the present study provides critical baseline data on phenotypic resistance in *An. gambiae s.l.* populations in Zuba, where such entomological data were previously unavailable. By establishing resistance profiles to two commonly used pyrethroids: deltamethrin and permethrin, this study supports the WHO’s call for strengthened operational monitoring and contributes to national efforts to sustain the effectiveness of malaria interventions. Our findings are intended to inform local vector control programs, support evidence-based policy development, and contribute to broader regional efforts to reduce malaria burden in Nigeria and its surrounding areas. 

## METHODS

**Study Site:** Zuba (latitude 9.1023, longitude 7.1952), a bustling community in the Gwagwalada Area Council of the FCT, Abuja, is home to a diverse, growing population. Many residents are involved in small-scale trading, farming, and other informal jobs that reflect the semi-urban character^23^. Environmental conditions in Zuba, such as stagnant water from poor drainage, open waste disposal, and seasonal rainfall, create favorable breeding sites for mosquitoes. These factors, combined with the area's expanding population and unregulated housing development, contribute to the high risk of mosquito infestation, making Zuba a key location for studying malaria vectors and insecticide resistance.

**Collection and Rearing of Immature Mosquito:** *An. gambiae s.l*. larvae were collected in June 2024 from temporary rain puddles and abandoned vehicle tires in Zuba, FCT, Nigeria. Larval sampling was conducted following standard procedures^24^ ^,^ ^25^. Larvae were collected by lightly submerging white dippers at a 45° angle into the breeding sites, skimming the water’s surface, and subsequently transferring the samples into labeled bottles for collection. Subsequently, the collected larvae were transported to the laboratory of the Center for Malaria Control and Neglected Tropical Diseases (CMC-NTDs), Suleja, Nigeria. In the insectary, larvae were reared under regulated conditions (temperature: 25-28 °C, humidity: approximately 70-80%, and a 12-hour light/dark cycle) and fed daily with yeast. Upon emergence, adult mosquitoes were supplied with a 10% glucose solution absorbed in cotton wool^26^.

**Preparation of Recommended and Lower Concentrations of Insecticides:** A 1 ml aliquot each of technical-grade stock solution of permethrin and deltamethrin, provided by the CDC, was diluted with 49 ml and 28.5 ml of 100% acetone, to prepare standard concentration of 21.50 μg/ml (permethrin) and 12.50 μg/ml (deltamethrin). These concentrations were used to coat CDC bottles with a capacity of 25 ml, resulting in the recommended concentrations of 21.50 μg/bottle (μg/b) for permethrin and 12.50 μg/b for deltamethrin^22^ ^,^ ^27^.

Lower concentrations of permethrin and deltamethrin were prepared in accordance with established protocols^28^, yielding 1 ml aliquots at the following concentrations: 15, 5, 1, 0.9, 0.8, 0.6, 0.4, 0.2, and 0 μg/b (control, acetone only). This process is essential for evaluating the lethal and sub-lethal effects of insecticides on exposed mosquitoes. A summary of all prepared dilutions is provided in Supplementary Materials 1 and 2.

**SUPPLEMENTARY MATERIAL1:** Serial dilutions of permethrin and the corresponding volumes of stock solution and acetone used in the CDC bottle bioassay.

Serial No.	Concentration (µg/bottle)	Volume of stock taken (mL)	Volume of acetone (mL)
1	15.0	0.9800	0.0200
2	10.0	0.8000	0.2000
3	5.0	0.4000	0.6000
4	1.0	0.0800	0.9200
5	0.9	0.0720	0.9280
6	0.8	0.0640	0.9360
7	0.6	0.0480	0.9520
8	0.4	0.0320	0.9680
9	0.2	0.0160	0.9840
Control	0.0	-	1.0000

**Note:** Stock solution and acetone were mixed to achieve the desired permethrin concentration in each CDC bottle. The control group was administered acetone alone.

**SUPPLEMENTARY MATERIAL 2:** Serial dilutions of deltamethrin and the corresponding volumes of stock solution and acetone used in the CDC bottle bioassay.

Serial No.	Concentration (µg/bottle)	Volume of stock taken (mL)	Volume of acetone (mL)
1	10.0	0.8000	0.2000
2	5.0	0.4000	0.6000
3	1.0	0.0800	0.9200
4	0.9	0.0720	0.9280
5	0.8	0.0640	0.9360
6	0.6	0.0480	0.9520
7	0.4	0.0320	0.9680
8	0.2	0.0160	0.9840
Control	0.0	-	1.0000

**Note:** Stock solution and acetone were mixed to achieve the desired deltamethrin concentration in each CDC bottle. The control group was administered acetone alone.

**Mosquito Exposure to Permethrin and Deltamethrin Insecticides:** Adult mosquitoes were maintained under controlled insectary conditions at 26 ± 2 °C, 75 ± 5% relative humidity, and a 12:12 h light:dark photoperiod. To evaluate the lethal and sub-lethal effects of permethrin and deltamethrin, mosquitoes were exposed to both the WHO-recommended and sub-lethal concentrations of each insecticide. Each concentration was used to coat clean, dry, and labeled CDC bottles with a capacity of 25 ml. Subsequently, 25 adult mosquito samples were introduced into each bottle in four replicates, including a control, following standard procedure^20^ ^,^ ^28^. Mosquitoes were exposed to insecticides in CDC bottles for 30 min and then transferred to holding cages for observation. Mortality was recorded continuously from the time of exposure up to 24 h post-exposure.

**Statistical Analysis:** Mosquito mortality data were analyzed using IBM-SPSS statistics software version 25.0 and presented as mean mortality ± standard deviation (SD). Differences among insecticide concentrations were assessed using one-way analysis of variance (ANOVA), followed by Tukey’s Honestly Significant Difference (HSD) post-hoc test, where applicable, to determine pairwise differences between treatment means. All analyses were performed at a significance level of P ˂ 0.05. Graphs were generated using GraphPad Prism version 8. 

**Ethics statement:** This research received ethical clearance from the CMC-NTDs located in Suleja, Nigeria. The proposal was carefully reviewed and approved by the Center’s Ethical Review Committee to ensure that all procedures were aligned with national research ethics and international standards. Ethical approval for the study was granted under reference number CMC-NTDs/EC/2024/014. Prior to field and laboratory activities, informed consent protocols were followed, where required. All mosquito collection and handling procedures were performed in strict adherence to established safety and ethical guidelines.

## RESULTS

The recommended concentration of permethrin (21.5 µg/b), as specified by the CDC, did not achieve 100% mortality of adult *An. gambiae s.l.* within 30 min of exposure (Table 1). At this recommended concentration, a mean mortality rate of 21.10±0.50 (84.4%) was achieved, which was significantly higher (*P* = 0.031) than 18.00±0.00 (72%) mortality rate recorded at a lower concentration (15.0 µg/b) of permethrin. Complete mortality was only attained after 35 min at the recommended concentration (21.5 µg/b) of permethrin. These results indicated a decline in mosquito mortality with decreasing concentrations of permethrin. 

**TABLE 1:** Mortality response of *An. gambiae s.l.* to varying lethal concentrations of permethrin over time in Zuba, FCT, Nigeria.

Time (min)	21.5 µg/b	15.0 µg/b	10.0 µg/b	5.0 µg/b	1.0 µg/b	0.9 µg/b
0	-	-	-	-	-	-
15	15.00 ± 1.89ᵇ (60%)	10.00 ± 0.58ᵃ (40%)	7.00 ± 1.51ᵃ (28%)	-	-	-
30	21.10 ± 0.50ᶜ (84.4%)	18.00 ± 0.00ᵇ (72%)	7.00 ± 1.51ᵃ (28%)	-	-	-
35	25.00 ± 0.00ᶜ (100%)	21.00 ± 0.50ᶜ (84%)	10.00 ± 0.58ᵃ (40%)	1.00 ± 0.50ᵃ (4%)	-	-
40	25.00 ± 0.00ᶜ (100%)	24.80 ± 2.22ᶜ (99.2%)	21.00 ± 0.50ᶜ (84%)	1.00 ± 0.50ᵃ (4%)	-	-
45	25.00 ± 0.00ᶜ (100%)	25.00 ± 0.00ᶜ (100%)	21.00 ± 0.50ᶜ (84%)	4.00 ± 0.83ᵃ (16%)	-	-
60	25.00 ± 0.00ᶜ (100%)	25.00 ± 0.00ᶜ (100%)	25.00 ± 0.00ᶜ (100%)	23.00 ± 1.71ᶜ (92%)	15.00 ± 1.89ᵇ (60%)	-
24 h	25.00 ± 0.00ᶜ (100%)	25.00 ± 0.00ᶜ (100%)	25.00 ± 0.00ᶜ (100%)	25.00 ± 0.00ᶜ (100%)	15.00 ± 1.89ᵇ (60%)	1.00 ± 0.50ᵃ (4%)

**Note:** Values represent mean mortality ± SD), with percentage mortality shown in parentheses; superscripts with different letters within the same row indicate statistically significant differences at *P < 0.05* (n = 25), while identical letters indicate no significant difference at *P < 0.05* (n = 25); μg/b refers to micrograms per 25-ml CDC bottle; and “**-”** denotes 0.00 ± 0.00^a^ (0), indicating zero mean mortality ± SD) and zero percentage mortality.

**FIGURE 1:**
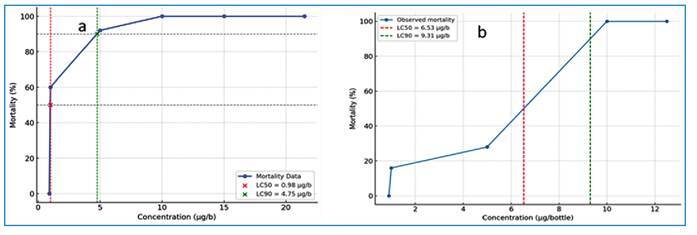
Concentration-mortality response of *An. gambiae s.l.* to: (a) permethrin after 60 min of exposure, and (b) deltamethrin after 60 min of exposure.

Note: LC_50_ (50% mortality) ≈ 0.98 µg/b; LC_90_ (90% mortality) ≈ 4.75 µg/b in (a), and LC_50_ (50% mortality) ≈ 6.53 µg/b; LC_90_ (90% mortality) ≈ 9.31 µg/b in (b).

Notably, *An. gambiae s.l.* population in Zuba appears to exhibit potential resistance to the CDC-recommended concentration of permethrin, with a sharp increase in mortality rate after 60 min of exposure, from 0% at 0.9 µg/b and 60% at 1.0 µg/b to complete (100%) mortality at concentrations ≥10.0 µg/b **(** Figure 1a).

The CDC-recommended concentration of deltamethrin (12.5 µg/b) did not achieve 100% mortality in adult *An. gambiae s.l.* within 30 min of exposure (Table 2). Complete mortality was achieved after 40 min of exposure to this concentration. This finding indicated that mosquito mortality rates increased with prolonged exposure to various concentrations of deltamethrin. In particular, the mean mortality rate of 19.00±0.30 (76.0%) was observed after 30 min of exposure to 12.5 µg/b deltamethrin. In contrast, a significantly higher (P = 0.04) mean mortality rate of 25.00±0.00 (100%) was recorded after 40 min of exposure to the same concentration. At a lower concentration of 0.9 µg/b, minimal lethality (3% mortality) was observed after 24 h of exposure, suggesting a threshold effect. The estimated LC₅₀ and LC₉₀ values were 6.53 µg/b and 9.31 µg/b, respectively, highlighting moderate resistance in the tested population (Figure 1b). 

**TABLE 2:** Mortality response of *An. gambiae s.l.* to varying lethal concentrations of deltamethrin over time in Zuba, FCT, Nigeria.

Time (min)	12.5 µg/b	10.0 µg/b	5.0 µg/b	1.0 µg/b	0.9 µg/b
0	-	-	-	-	-
15	15.00 ± 1.89ᵇ (60)	7.00 ± 1.51ᵃ (28)	-	-	-
30	19.00 ± 0.30ᵇ (76.0)	7.00 ± 1.51ᵃ (28)	-	-	-
35	21.10 ± 0.50ᶜ (84.4)	10.00 ± 0.58ᵃ (40)	-	-	-
40	25.00 ± 0.00ᶜ (100)	21.00 ± 0.50ᵇ (84)	1.00 ± 0.50ᵃ (4)	-	-
45	25.00 ± 0.00ᶜ (100)	21.00 ± 0.50ᵇ (84)	1.00 ± 0.50ᵃ (4)	-	-
60	25.00 ± 0.00ᶜ (100)	25.00 ± 0.00ᶜ (100)	7.00 ± 0.83ᵃ (28)	4.00 ± 1.89ᵃ (16)	-
24 h	25.00 ± 0.00ᶜ (100)	25.00 ± 0.00ᶜ (100)	20.00 ± 0.00ᶜ (80)	10.00 ± 0.58ᵃ (40)	0.75 ± 0.20ᵃ (3)

Note: Values represent mean mortality ± SD, with percentage mortality shown in parentheses; superscripts with different letters within the same row indicate statistically significant differences at *P < 0.05* (n = 25), while identical letters indicate no significant difference at *P < 0.05* (n = 25); μg/b refers to micrograms per 25-ml CDC bottle; and “-"” denotes 0.00 ± 0.00^a^ (0), indicating zero mean mortality ± SD and zero percentage mortality.

Mean mortality rate of 0.00±0.00 (0%) was recorded for adult *An. gambiae s.l.* exposed to sub-lethal concentrations of permethrin and deltamethrin (0.8, 0.6, 0.4, 0.2, and 0.0 µg/b) for 24 h. These results were not significantly different from those of the control group (*P* = 0.061) (Table 3). These results confirmed that the tested concentrations were ineffective against *An. gambiae s.l*. population in Zuba, as no observable mortality or delayed toxic effects were recorded within 24 h.

**TABLE 3:** Mean mortality of *An. gambiae s.l.* in Zuba, FCT, Nigeria when exposed to sub-lethal concentrations of permethrin and deltamethrin.

Time (min)	0.8 µg/b	0.6 µg/b	0.4 µg/b	0.2 µg/b	0.0 µg/b (Control)
0	-	-	-	-	-
15	-	-	-	-	-
30	-	-	-	-	-
35	-	-	-	-	-
40	-	-	-	-	-
45	-	-	-	-	-
60	-	-	-	-	-
24 h	-	-	-	-	-

“-" denote 0.00 ± 0.00^a^ (0%), indicating zero mean mortality ± SD and zero percentage mortality.

## DISCUSSION

The WHO has developed criteria for evaluating pesticide resistance in mosquito populations. According to these guidelines, a mosquito population is considered resistant when less than 80% mortality is observed following exposure to a WHO-approved insecticide concentration, using the 30-min exposure protocol of the CDC bottle bioassay. Mortality rates between 98% and 100% indicated susceptibility, whereas rates between 80% and 97% suggested possible or suspected resistance^2^ ^,^ ^7^ ^,^ ^25^ ^,^ ^26^. 

The mortality rate of *An. gambiae s.l.* vectors in the study area was 84.4% for permethrin and 76% for deltamethrin following exposure to the WHO-recommended concentrations, both falling below the WHO’s 98% threshold for confirmed susceptibility. These findings indicate operationally significant resistance to both insecticides and reflect a broader trend across Nigeria, attributed to the historical overreliance on pyrethroid-based vector control interventions. Since the early 2000s, the country’s malaria control strategies have heavily relied on the distribution of ITNs and the implementation of IRS, both of which predominantly utilize pyrethroids^7^. This prolonged and widespread use has exerted selective pressure on mosquito populations, facilitating the emergence and spread of resistance mechanisms, particularly those associated with metabolic resistance pathways^10^ ^,^ ^18^. 

The observed resistance of *An. gambiae s.l.* to deltamethrin and the suspected resistance to permethrin in Zuba highlight the growing challenge of insecticide resistance in malaria-endemic regions. Although pyrethroid resistance has been documented in several parts of Nigeria^12^ ^-^ ^14^, this study, to the best of our knowledge, provides the first phenotypic assessment of pyrethroid susceptibility in *An. gambiae s.l.* population in Zuba, a locality for which entomological surveillance data are lacking. This localized evidence is particularly important in light of the WHO’s emphasis on the significance of district- and country-level resistance monitoring as a foundation for informed context-specific vector control strategies. By establishing baseline data for this area, our findings offer a valuable reference point for future resistance surveillance, enabling comparisons with other regions and guiding the development of future resistance management strategies in the area. 

The resistance levels reported in *An. gambiae s.l.* population in this study are likely driven by a combination of environmental and biological factors. In addition to the prolonged and widespread use of pyrethroids in IRS, their concurrent application in agricultural pest control may have exerted sustained selective pressure on local mosquito populations, favoring the survival and propagation of resistant genotypes. Consequently, mosquitoes carrying genetic mutations or traits that confer resistance to pyrethroids are more likely to survive and reproduce successfully under selective pressure^29^. Metabolic resistance mechanisms, such as the overexpression of detoxifying enzymes, including cytochrome P450s, glutathione S-transferases, and esterases, may further reduce the effectiveness of insecticides through breakdown of active compounds before they reach their target sites^25^ ^,^ ^30^. Additionally, target-site mutations, particularly knockdown resistance (*kdr*) mutations in the voltage-gated sodium channel gene, have been widely associated with reduced pyrethroid sensitivity in *An. gambiae s.l.* population^31^. Notably, a previous study across northern Nigeria reported moderate to high levels of resistance to deltamethrin and permethrin in wild mosquito populations, which was largely attributed to similar selective pressure^32^. Our findings in Zuba are consistent with these earlier observations but provide a critical update for a distinct and previously uncharacterized geographical zone within the FCT for regional malaria planning. 

Lethal concentration, the amount of an insecticide required to cause mortality in a specific proportion of an exposed population, commonly expressed as LC_50_ and LC_90_ and represent the concentration causing mortality in 50% and 90% of exposed individuals, respectively^5^ ^,^ ^33^ ^-^ ^35^. In contrast, sub-lethal concentrations do not cause immediate mortality but may induce physiological or behavioral alterations that compromise long-term survival, reproduction, or development. These effects include impaired locomotion, feeding inhibition, hormonal disruption, or reduced fertility^28^. Notably, repeated exposure to sub-lethal concentrations of insecticides has been identified as a key driver of insecticide resistance in mosquito populations, as it selectively favors individuals possessing traits that enhance survival under chemical stress^18^ ^,^ ^25^ ^,^ ^36^.

The present findings indicate that deltamethrin concentrations of 12.5, 15, 5, 1, and 0.9 μg/b induced varying degrees of mortality in field-collected *An. gambiae s.l.* population in Zuba, which is consistent with a report by Adesoye *et al.* ^27^, who observed similar lethality in *An. gambiae s.l*. (Kisumu) strain when exposed to equivalent permethrin concentrations. Similar resistance trends have been documented in Jigawa and other northern states, where wild *An. gambiae s.l.* populations exhibited moderate to high resistance to the WHO-recommended pyrethroid concentrations^19^ ^,^ ^34^. Comparative studies using susceptible Kisumu strain of *An. gambiae s.l.* demonstrated higher mortality even at lower permethrin concentrations, highlighting the marked resistance observed in wild mosquito populations, such as those in Zuba^27^. These findings reinforce the need to implement rotational insecticide use with alternative modes of action and integrate robust resistance management strategies into Nigeria’s malaria vector control framework.

In contrast, the findings of another study^27^ differ from those of the present study, as only 0.2 and 0.4 μg/b permethrin concentrations failed to induce any mortality at the 24-h mark, in contrast to our research. However, in the present study, concentrations of 0.8, 0.6, 0.4, and 0.2 μg/b produced mortality rates that were not significantly different from the control experiment (0%) after 24 h, rendering all these doses sub-lethal for the mosquito population. This discrepancy may be attributed to the use of wild mosquitoes in the current study, rather than the well-established susceptible *An. gambiae s.l.* (Kisumu) strain used in the previous study.

Our findings provide compelling evidence that *An. gambiae s.l.* population in Zuba, FCT, Nigeria, has developed resistance to deltamethrin and shows suspected resistance to permethrin, despite both being recommended concentrations by the CDC. These results highlight a trend indicating the reduced effectiveness of recommended insecticide concentrations, which poses a direct threat to the success of current malaria vector control strategies, such as ITNs and IRS.

This resistance may contribute to sustained malaria transmission in the region, even in the presence of ongoing vector control efforts. Therefore, malaria control programs in Nigeria must incorporate routine insecticide resistance monitoring and adjust intervention strategies accordingly. This may include the integration of alternative insecticides, deployment of synergist-based nets, or rotation of active compounds to delay further development of resistance.

However, this study has some limitations. Data were collected from a single location, Zuba, the Gwagwalada Area Council, which may limit the generalizability of the findings across other ecological zones in Nigeria. In addition, although phenotypic resistance was observed through mortality assays, the study did not evaluate the specific resistance mechanisms, such as metabolic resistance, target- site mutations, or behavioral avoidance, that contribute to reduced susceptibility. Therefore, future studies incorporating molecular and biochemical analyses are recommended to better understand the underlying drivers of resistance and inform targeted interventions. 

In conclusion, although resistance to deltamethrin and permethrin is not unprecedented in Nigeria, this study is the first to address a critical geographical and temporal data gap by documenting insecticide resistance in Zuba. These findings highlight the need for routine, localized resistance monitoring and adaptive malaria vector control strategies, including diversifying insecticide options, deploying synergist-based interventions, and integrating non-pyrethroid compounds, to sustain the effectiveness of vector control measures and mitigate malaria transmission risks in the region.

## Data Availability

The underlying data for this study can be accessed by contacting the corresponding author
